# HIV patients admitted to an ICU of a university hospital - experience of 15 years: 1995 to 2009

**DOI:** 10.1186/cc12404

**Published:** 2013-03-19

**Authors:** A Agrifoglio, M Arce, M Hernández, P Millan, C Guallar, I Pozuelo, M Jiménez

**Affiliations:** 1 Hospital Universitario La Paz, Madrid, Spain

## Introduction

HIV infection is a major public health problem in the world. The use of prophylaxis against opportunist infection and the introduction of HAART in 1996 increased life expectancies. The therapeutic use of ICU resources for HIV patients has been controversial, questioning the admission of these patients especially in advanced stages of the disease, given the poor prognosis. The aim of this study was to determine the experience of the past 15 years in relation to the income of these patients in an ICU.

## Methods

A retrospective case series consisting of patients with diagnosis of HIV infection (known or unknown) admitted between January 1995 and December 2009. We collected demographic and epidemiological data, process of acquisition of the disease, infection status: known or unknown patient infected, whether or not receiving antiretroviral therapy and whether it was effective (undetectable viral load at the time of admission), APACHE II, cause of admission, need for mechanical ventilation (MV), pathology related or unrelated to HIV infection and ICU mortality.

## Results

During this period 12,607 patients were admitted to the ICU, 188 (1.5%) HIV-positive. Mean APACHE II score 17.6, median age 39 years, 73% men and 90% Spanish nationality. Principal risk behavior: addiction drugs injection (67%). Seventeen percent of patients did not know who was infected with HIV at the time of admission to the ICU. Fifty-three percent were not receiving HAART. Of the 88 patients treated, 93% were receiving HAART (effective in 88% of cases). Sixty percent of the patients came from the emergency department of the hospital. Main admission diagnoses: acute respiratory failure caused by infection (*Streptococcus pneumoniae *and *Pneumocystis jirovecii*), neurological disorders (coma for illicit drugs and psychotropic) and septic shock. Seventy percent required MV. Of patients whose HIV infection was not known, 93.5% were admitted for related pathology. In patients of known infection, the pathology associated with HIV was 30%. Average length of stay 9 was days. ICU mortality was 35%. Most frequent causes of death: septic shock and multiple organ failure.

## Conclusion

Depending on the patient and the cause of admission, ICU admission may represent an excellent opportunity as a screening method to determine HIV status. Given the greater efficacy of HAART at present, most patients with medical or surgical conditions unrelated to HIV infection will be eligible to join the ICU. People with HIV can and should benefit from using reasonable and individualized care in an ICU.
